# Proteomic analysis reveals potential factors associated with enhanced EPS production in *Streptococcus thermophilus* ASCC 1275

**DOI:** 10.1038/s41598-020-57665-9

**Published:** 2020-01-21

**Authors:** Aparna Padmanabhan, Yin Tong, Qinglong Wu, Clive Lo, Nagendra P. Shah

**Affiliations:** 1 School of Biological Sciences, The University of Hong Kong, Pokfulam Road, Hong Kong SAR, China; 2 Department of Pathology and Immunology & Texas Children’s Microbiome Center, Baylor College of Medicine, 1 Baylor Plaza, Houston, TX 77030 USA

**Keywords:** Microbiology, Proteomics

## Abstract

*Streptococcus thermophilus* ASCC 1275 has two chain length determining genes - *epsC* and *epsD*- in its *eps* gene cluster, and produces two times more EPS in sucrose medium than that in glucose and lactose. Hence, we investigated the influence of sugars (glucose, sucrose and lactose), at log phase (5 h) and stationary phase (10 h), on the global proteomics of *S. thermophilus* 1275 to understand the differentially expressed proteins (DEPs) during EPS production using isobaric tags for relative and absolute quantitation (iTRAQ)-based proteomic analysis. Among 98 DEPs in sucrose medium, most of them were mapped into EPS biosynthesis pathway and other related metabolisms. There was an upregulation of several proteins involved in sugar transport (phosphoenolpyruvate (PEP) phosphotransferase system), EPS assembly (*epsG1D*) and amino acid metabolism (methionine, cysteine/arginine metabolism) in sucrose medium. This study showed that increased EPS production in *S. thermophilus* 1275 requires a well-co-ordinated regulation of pathway involved in both EPS assembly and amino acid metabolism along with the availability of sugars. Thus, it provided valuable insights into the biosynthesis and regulation of EPS in *S. thermophilus* 1275, and potential gene targets for understanding high-EPS strains.

## Introduction

Exopolysaccharides (EPS) are long polymeric chain of carbohydrates usually synthesised by various microorganisms including bacteria, fungi, microalgae as well as plants^1,2^. Among bacteria, the EPS produced from lactic acid bacteria (LAB) is of great importance due to the general acceptability of LAB in fermented food production and functional attributes of the EPS produced by LAB^3^. In food industry, EPS produced from LAB starter cultures have been used as moisture retention agents in cheese to improve its functionality^1,4^; as bio-thickeners in yogurt to improve its mouthfeel, texture and to avoid syneresis^5–7^. It has also been traditionally used to produce fermented drinks like viili and kefir. Besides, EPS from LAB has the potential to replace synthetically modified plant and algal polysaccharides in sectors like pharmaceutics, medicine, and cosmetics. However, the low yield and high cost of production are the main limiting factors for the commercial exploitation of EPS from LAB. To overcome these issues, strategies like screening of high EPS producing stains, optimization of fermentation conditions, and use of cheap substrates have been adopted. However, the global pathway analysis of existing EPS producing strains using emerging omics techniques like proteomics, and further targeting specific genes to produce excess EPS is found to be promising to increase EPS yield^8^.

Among LAB*, Streptococcus thermophilus* is a conventional dairy starter bacterium which has a huge market in the dairy industry^9^. It is a non-pathogenic, homofermentative facultative anaerobe and is widely used in the production of fermented dairy foods like yogurt and cheese in combination with *Lactobacillus delbrueckii* ssp. *bulgaricus*^10,11^. *S. thermophilus* plays a vital role in the fast pH reduction of milk by producing lactic acid and imparts flavour to fermented foods. Some strains of *S. thermophilus* are known to produce exopolysaccharides (EPS) that can improve the texture and viscosity of fermented dairy foods. Our previous study showed that *S. thermophilus* ASCC 1275 can produce high amount of EPS (~1 g/L) in milk supplemented with 0.5% whey protein concentrate (WPC) when compared with other *S. thermophilus* strains^12^. It was also found to produce two types of EPS - capsular and ropy EPS. Due to the presence of ropy EPS, *S. thermophilus* 1275 could enhance the texture of yogurt and Mozzarella cheese. The whole genome sequencing of *S. thermophilus* 1275 revealed that it has a unique set of chain length determining genes in its EPS gene cluster when compared with the other five fully sequenced *S. thermophilus* strains^10^. We have also observed that the sugar available in the media and growth phase influence the amount of EPS produced by *S. thermophilus* 1275 and the genes that lead to the production of EPS^13^. Another interesting feature of *S. thermophilus* 1275 is the presence of an effective proteolytic system with several intracellular peptidases and proteases^10^. A rarely found extracellular proteinase PrtS that cleaves casein to oligo-peptides is also present in *S. thermophilus* 1275^14^. Hence, it would be interesting to understand the global level proteomic changes occurring in this high EPS producing bacterium in the presence of various sugars, which can highly influence EPS production in *S. thermophilus* 1275.

Based on our previous study, sucrose (1%) was found to produce more EPS in M17 medium (~430 mg/L) at stationary phase (12 h) when compared to glucose (~276 mg/L) and lactose (~163 mg/L) at the same concentration^13^. This significant variation in EPS production with different sugars motivated us to understand the changes at gene level that may be occurring in *S. thermophilus* 1275. The study on the genomic insights of *S. thermophilus* 1275 provided a well-documented database for transcriptomics and proteomics analysis^10^. Moreover, our recent transcriptomics study on *S. thermophilus* 1275 under three different sugars and two growth phases provided information about the differentially expressed genes mainly related to EPS production^13^. In this study, the strain *S. thermophilus* 1275 was used to understand the global level proteomic changes influenced by three selected sugars (glucose, sucrose and lactose) and two growth phases (log phase, 5 h; stationary phase,10 h). Proteomics study would provide information about proteins that directly function in the cell and are closer to the operational level^15^. Recently, iTRAQ based quantitative proteomic analysis was found to provide proteome profiles with high robustness and resolution. Hence, in this study we employed iTRAQ analysis to identify the differentially expressed proteins in *S. thermophilus* 1275 influenced by sugars and growth phases. Furthermore, functional classification and pathway enrichment analysis of DEPs were done using clusters of orthologous groups (COG) and Kyoto Encyclopedia of Genes and Genomes (KEGG) analysis.

## Results

### Protein identification

**Figure 1:**
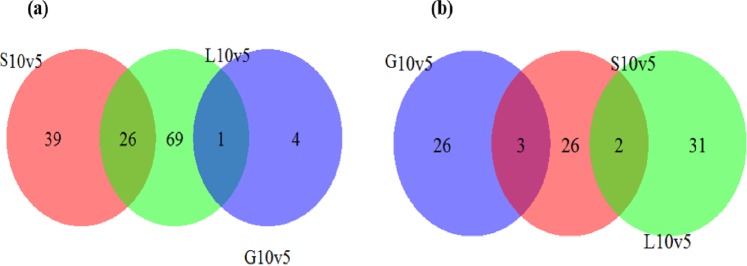
Venn diagram comparing the common up-regulated and down-regulated proteins in each condition. DEPs identified in all sugars when 10 h was compared with 5 h (**a**) DEPs upregulated (**b**) DEPs downregulated.

Quantitative proteomic analysis using iTRAQ labelling method was performed to profile the expression differentially expressed proteins in *S. thermophilus* 1275 in the presence of glucose, sucrose and lactose at 5 h and 10 h. Triplicate protein samples were collected from M17-G, M17-S and M17-L at two time points to ensure biological reproducibility. iTRAQ labels 113, 114, 115, 116, 117, 118 were separately used to label samples from M17-G (5 h), M17-G (10 h), M17-S (5 h), M17-S (10 h), M17-L (5 h) and M17-L (10 h), respectively. A total of 16624 unique peptides related to 1027 proteins were identified, out of which 924 proteins (89.97%) had at least 2 unique peptides detected with 95% confidence and unused ProtScore higher than 1.28 (critical FDR 1%), which were the two analysis thresholds used in this study. Figure 1 and Supplementary Table S1 shows the number of DEPs identified in each sugar when stationary phase (10 h) was compared with lag phase (5 h). Total DEPs detected were 259, of which 166 proteins were upregulated and 93 proteins were downregulated in all sugars (Fig. 1). Twenty-six common proteins were upregulated in sucrose medium and lactose medium whereas only one common protein was upregulated in glucose medium and lactose medium (Fig. 1a, Supplementary Table S2). However, only a smaller number of common downregulated proteins, 3 and 2, were observed between glucose medium and sucrose medium as well as sucrose medium and lactose medium, respectively (Fig. 1b, Supplementary Table S2).

### Functional classification of DEPs based on COG and KEGG

**Figure 2:**
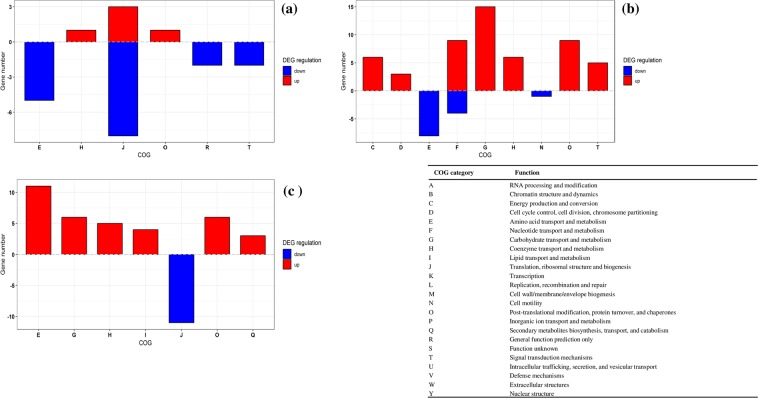
COG classification of DEPs in different sugars (**a**) glucose G10h Vs G5h (**b**) lactose L10h Vs L5h (**c**) sucrose S10h Vs S5h. Red and blue bar indicate the number of upregulated and down regulated proteins, respectively.

**Figure 3:**
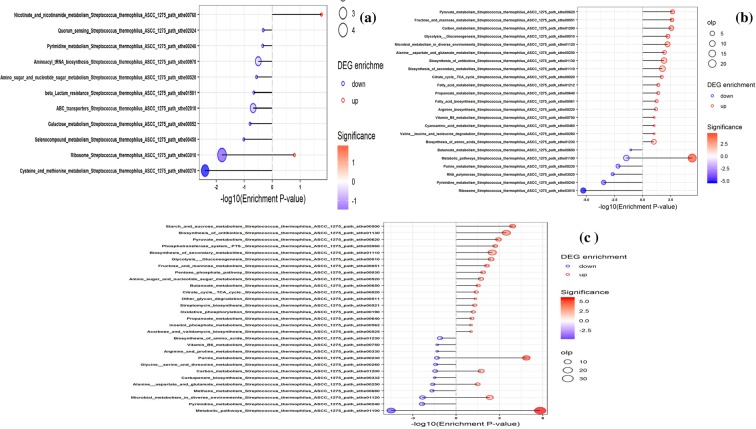
KEGG classification of DEPs in different sugars (**a**)glucose G10h Vs G5h (**b**) sucrose S10h Vs S5h (**c**) lactose L10h Vs L5h. Red and blue bar indicate the number of upregulated and down regulated proteins, respectively.

The functional classification of total identified (DEP) proteins were performed using COG and KEGG analysis (Figs. 2 and 3). According to COG database 676 proteins were classified into 20 categories. DEPs from 10 h was compared with 5 h in glucose-, sucrose- and lactose media. In M17-G, proteins were mainly down-regulated in the categories translation, ribosomal structure and biogenesis- J, amino acid transport and metabolism- E, signal transduction mechanisms- T, general function prediction only- R and up-regulated in the categories post-translational modification, protein turnover, and chaperones- O, coenzyme transport and metabolism- H, translation, ribosomal structure and biogenesis- J (Fig. 2a, Supplementary Tables S3 and S4). In M17-L down regulation was observed in proteins involved in the categories amino acid transport and metabolism- E, nucleotide transport and metabolism - F, Cell motility- N and up-regulation were observed in categories Carbohydrate transport and metabolism- G, Nucleotide transport and metabolism- F, Post-translational modification, protein turnover, and chaperones- O, Coenzyme transport and metabolism- H, Energy production and conversion- C, Signal transduction mechanisms- T, Cell cycle control, cell division, chromosome partitioning- D (Fig. 2b, Supplementary Tables S3 and S4). Similarly, in M17-S, proteins in the category Translation, ribosomal structure and biogenesis- J was down regulated and proteins in the categories Amino acid transport and metabolism- E, Carbohydrate transport and metabolism- G, Post-translational modification, protein turnover, and chaperones- O, Coenzyme transport and metabolism- H, Lipid transport and metabolism- I, Secondary metabolites biosynthesis, transport, and catabolism- Q were up-regulated (Fig. 2c, Supplementary Tables S3 and S4).

As per KEGG analysis, in M17-G, cysteine and methionine metabolism was significantly down regulated along with ribosomal RNAs and ribosomal proteins, seleno-compound metabolism, galactose metabolism, ABC transporters, β-lactum resistance, amino sugar and nucleotide metabolism, ABC transporters, aminoacyl tRNA biosynthesis, pyrimidine metabolism and quorum sensing. Up-regulation of proteins in glucose media was observed only in a few proteins involved in ribosomal RNAs and ribosomal proteins and nicotinate and nicotinamide metabolism (Fig. 3a, Supplementary Tables S5 and S6). When compared to glucose a greater number of pathways were found to get up-regulated in sucrose and lactose media with a significant up regulation in metabolic pathways. Other major pathways up-regulated in M17-S were pyruvate metabolism, fructose and mannose metabolism, carbon metabolism, glycolysis, microbial metabolism in diverse environment, alanine/aspartate/glutamate metabolism, biosynthesis of antibiotics, biosynthesis of secondary metabolites, citrate cycle, fatty acid metabolism, propanoate metabolism, fatty acid biosynthesis, arginine biosynthesis, vitamin B6 metabolism, cyanoamino acid metabolism, valine/leucine/isoleucine degradation and biosynthesis of amino acids. A significant down-regulation of DEPs in M17-S was observed in ribosome proteins along with purine and pyrimidine metabolism, RNA polymerase, butanoate metabolism and a few proteins involved in metabolic pathways (Fig. 3b, Supplementary Tables S5 and S6). In M17-L, a significant up-regulation of many proteins involved in purine metabolism and metabolic pathways was observed along with a few proteins involved in the same pathways down-regulated. The other pathways up-regulated were sucrose metabolism, biosynthesis of antibiotics, pyruvate metabolism, PTS system, biosynthesis of secondary metabolites, glycolysis, fructose and mannose metabolism, pentose phosphate pathway, amino sugar and nucleotide sugar metabolism, butanoate metabolism, citrate cycle, other glycan degradation, streptomycin biosynthesis, oxidative phosphorylation, propanoate metabolism, inositol phosphate metabolism, purine metabolism, carbon metabolism, alanine aspartate and glutamate metabolism, and down regulated pathways were pyrimidine metabolism, methane metabolism, carbapenem biosynthesis, glycine/serine/threonine metabolism, arginine and proline metabolism, vitamin B6 metabolism, biosynthesis of amino acids and a few proteins involved in carbon metabolism, microbial metabolism, alanine/aspartate and glutamate metabolism, purine metabolism, glycine/serine/threonine metabolism (Fig. 3c, Supplementary Tables S5 and S6).

### Proteomic data validation using RT-qPCR

The validation of proteomic data was performed using RT-qPCR assay with eight selected genes with significantly different expression profile. As shown in Supplementary Table S7, all the genes selected were those involved in EPS biosynthesis. The trend of expression changes was consistent in both experiments with minor difference in fold change levels. This indicates that the proteomics results and RT-qPCR results were consistent, which could reflect the changes occurring in EPS biosynthesis of *S. thermophilus* 1275 in the presence of different sugars at two-time points.

### Growth associated changes in EPS biosynthesis related genes

Biosynthesis of EPS is a very complex process that requires the participation of various genes and proteins. Laws *et al*.^16^ classified the EPS biosynthesis into four major steps which involve 1) sugar intake into bacterial cell 2) synthesis of sugar-1-phosphate 3) EPS polymerization, and 4) transport of EPS outside the bacterial cell. To understand the EPS biosynthesis in *S. thermophilus* 1275, the DEPs involved in each step were analysed.

### Sugar intake in *S. thermophilus* 1275

**Figure 4:**
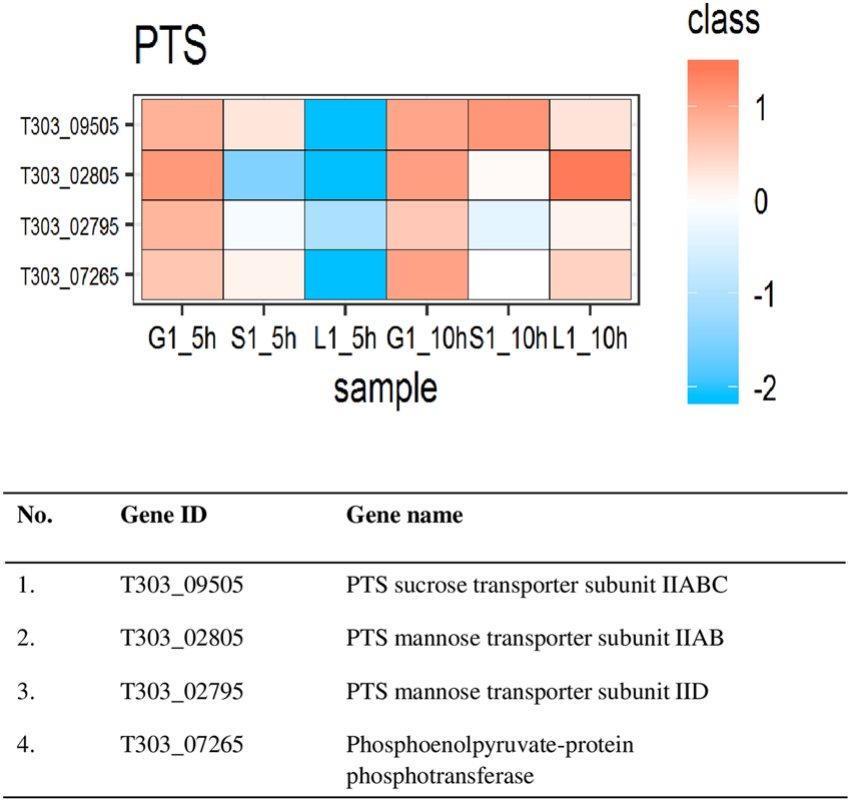
DEPs involved in PTS transport in different sugars at two-time points. Heat map of DEPs in PTS transport system under the influence of glucose (G), sucrose (S) and Lactose (L) at the 5 h and 10 h. Red and blue box indicate up-regulated and down-regulated proteins, respectively.

From the KEGG pathway map of *S. thermophilus* 1275 it was observed that the sugar transport system in this bacterium is through phosphotransferase (PTS) system. Hence, the DEPs in *S. thermophilus* 1275 PTS system responsible for glucose, sucrose and lactose were analysed (Fig. 4). At both the time points in M17-G, PTS mannose transporter subunit IID, PTS mannose transporter subunit IIAB, Phosphoenolpyruvate-protein phosphotransferase, PTS sucrose transporter subunit IIABC were active (Supplementary Table S1). In M17-S a significant up-regulation was observed in PTS sucrose transporter subunit IIABC at 10 h (Table 1). However, in M17-L, PTS sucrose transporter subunit IIABC, PTS mannose transporter subunit IIAB, PTS mannose transporter subunit IID, and Phosphoenolpyruvate-protein phosphotransferase were highly up-regulated at 10 h compared to 5 h (Supplementary Table S1).

**Table 1:** Differentially expressed proteins associated with EPS production in *S. thermophilus* 1275 in the presence of sucrose (10 h Vs 5 h).

Category	Protein description	Locus tag	Fold change	p-value	Regulation type
EPS assembly	eps1C	T303_06410	−1.61	0.046	DOWN
	epsE	T303_06400	−1.65	0.053	DOWN
Arginine, cysteine, methionine	Glutamate–cysteine ligase	T303_07930	2.36	0.040	UP
	Argininosuccinate synthase	T303_00025	−1.91	0.048	DOWN
	Urease subunit alpha	T303_02590	1.05	0.052	UP
	Glutamate dehydrogenase	T303_03260	2.15	0.069	UP
	Cysteine synthase	T303_02960	2.42	0.096	UP
Ribosomal proteins	50S ribosomal protein L22	T303_00600	−1.39	0.013	DOWN
	50S ribosomal protein L24	T303_00570	−0.62	0.014	DOWN
	rpsP; 30S ribosomal protein S16	T303_08560	−0.92	0.029	DOWN
	30S ribosomal protein S21	T303_08285	4.14	0.038	UP
	50S ribosomal protein L1	T303_00040	−1.16	0.038	DOWN
	glyQ; glycyl-tRNA synthase subunit alpha	T303_03630	1.75	0.040	UP
	50S ribosomal protein L4	T303_00620	−0.99	0.041	DOWN
	30S ribosomal protein S3	T303_00595	1.14	0.041	UP
	50S ribosomal protein L17	T303_00490	−1.23	0.042	DOWN
	rpsA; 30S ribosomal protein S1	T303_04125	−1.26	0.089	DOWN
	50S ribosomal protein L23	T303_00615	−0.66	0.094	DOWN
Carbohydrate metabolism	Acetyl-CoA carboxylase biotin carboxyl carrier protein subunit	T303_03105	0.70	0.003	UP
	6-phosphofructokinase	T303_03155	1.14	0.017	UP
	Dihydrolipoyl dehydrogenase	T303_06180	1.47	0.021	UP
	Glyceraldehyde-3-phosphate dehydrogenase	T303_09765	5.17	0.022	UP
	PTS mannose transporter subunit IIAB	T303_028052	1.56	0.039	UP
	Fructose-1,6-bisphosphate aldolase	T303_00465	3.05	0.040	UP
	Alpha-amylase	T303_08530	0.82	0.055	UP
	Pyruvate dehydrogenase E1 subunit alpha	T303_06195	0.59	0.056	UP
	Lactoylglutathione lyase	T303_08485	1.58	0.081	UP
	Formate acetyltransferase	T303_09120	−1.56	0.097	DOWN
Nucleotide sugar synthesis	PTS mannose transporter subunit IIAB	T303_02805	1.561	0.039	UP
	Glucosamine-fructose-6-phosphate aminotransferase	T303_05515	1.63	0.071	UP
Lipid metabolism	Acetyl-CoA carboxylase biotin carboxyl carrier protein subunit	T303_031051	0.70	0.003	UP
	UDP-N-acetylmuramoylalanyl-D-glutamate–2,6-diaminopimelate ligase	T303_02880	1.17	0.034	UP
	Peptidoglycan branched peptide synthesis protein	T303_04145	1.02	0.053	UP
	3-oxoacyl-ACP synthase	T303_03100	1.07	0.054	UP
	ACP S-malonyltransferase	T303_03090	−1.00	0.059	DOWN
Amino acid metabolism	Aspartate–ammonia ligase	T303_03050	1.24	0.016	UP
	Dihydrolipoyl dehydrogenase	T303_061801	1.47	0.021	UP
	Argininosuccinate synthase	T303_000251	−1.91	0.048	DOWN
	Glutamate dehydrogenase	T303_032601	2.15	0.070	UP
	Glucosamine-fructose-6-phosphate aminotransferase	T303_055151	1.63	0.071	UP
	Tryptophan synthase subunit beta	T303_08765	−0.72	0.080	DOWN
	Aminotransferase A	T303_01310	1.16	0.082	UP
	Ketol-acid reductoisomerase	T303_00290	0.60	0.089	UP
Glycan biosynthesis	UDP-N-acetylmuramoylalanyl-D-glutamate–2,6-diaminopimelate ligase	T303_028801	1.17	0.034	UP
	Peptidoglycan branched peptide synthesis protein	T303_041451	1.02	0.053	UP
Metabolism of cofactors vitamins	Purine nucleoside phosphorylase	T303_06430	−0.94	0.014	DOWN
	3-oxoacyl-ACP synthase	T303_031001	1.07	0.054	UP
	Ketol-acid reductoisomerase	T303_002901	0.60	0.089	UP
	Phosphomethylpyrimidine kinase	T303_01865	2.80	0.096	UP
Nucleotide metabolism	Dihydroorotate dehydrogenase	T303_05840	−2.96	0.002	DOWN
	Purine nucleoside phosphorylase	T303_064301	−0.94	0.014	DOWN
	Uracil phosphoribosyltransferase	T303_02910	2.37	0.015	UP
	Urease subunit alpha	T303_025901	1.05	0.052	UP
	Ribonucleoside triphosphate reductase	T303_00780	1.68	0.096	UP
Membrane transport	Serine protease	T303_01085	2.24	0.006	UP
	Peptide ABC transporter ATP-binding protein	T303_080751	−1.07	0.015	DOWN
	Glutamine ABC transporter substrate-binding protein	T303_066551	2.78	0.017	UP
	PTS mannose transporter subunit IIAB	T303_028053	1.56	0.039	UP
	Heme ABC transporter ATP-binding protein	T303_026951	−0.60	0.061	DOWN
	artP; arginine ABC transporter ATP-binding protein	T303_066501	3.37	0.081	UP
	Glutamine ABC transporter permease	T303_066601	2.09	0.092	UP
PTS	PTS mannose transporter subunit IIAB	T303_028051	1.56	0.038	UP

### Nucleotide sugar synthesis

**Figure 5:**
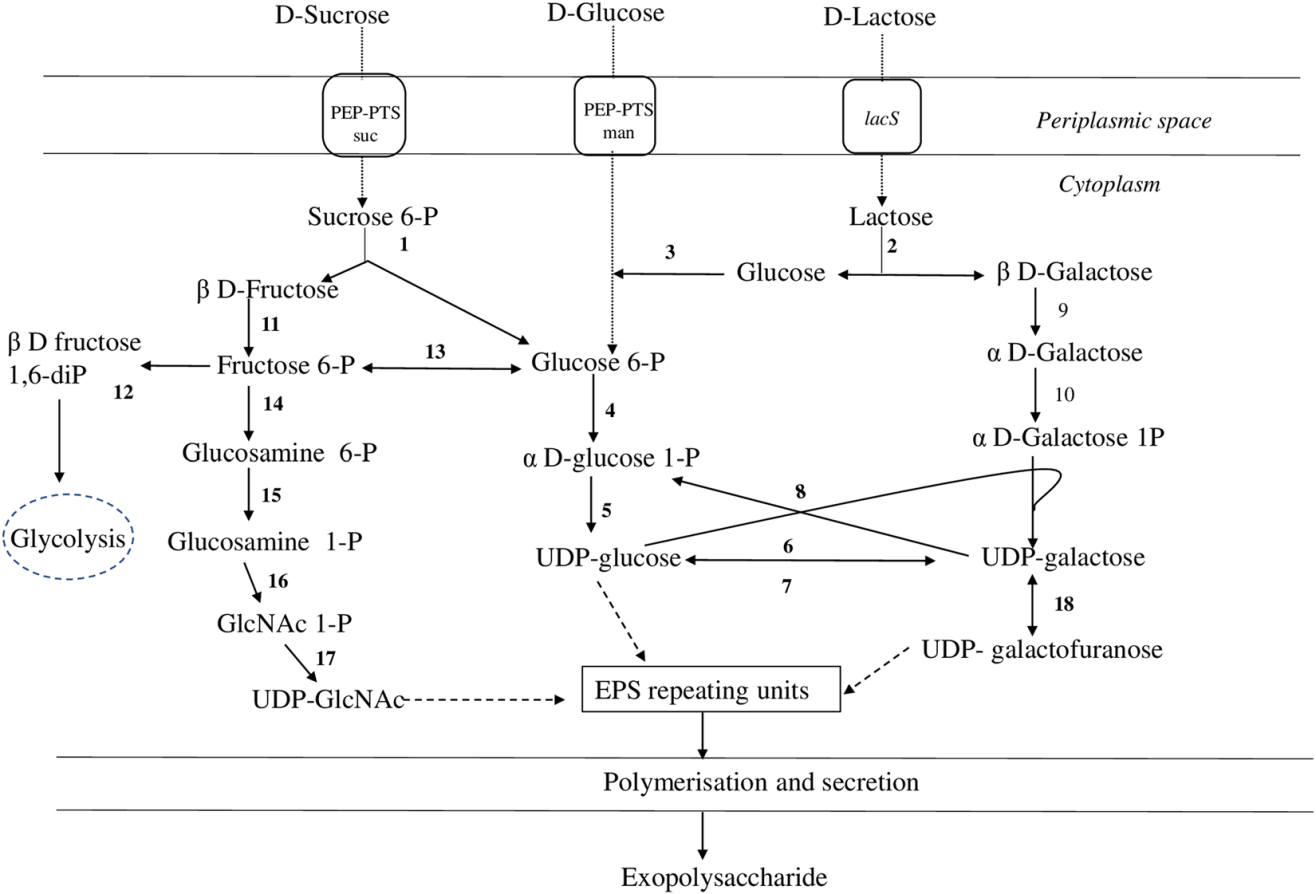
Pathway for EPS production in *S. thermophilus* 1275 in the presence of glucose, sucrose and lactose^13^. Enzymes are indicated by the numbers: (1) T303_09510 β-fructofuranosidase, (2) T303_07865 β-Galactosidase, (3) T303_04850 Glucokinase, (4) T303_05140 Phosphoglucomutase, (5) T303_00105 UDP-glucose pyrophosphorylase, (6) T303_07880 UDP-glucose 4-epimerase, (7) T303_06690 UDP-galactose-4-epimerase, (8) T303_07885 Galactose-1-phosphate uridylyltransferase, (9) T303_07875 Galactose mutarotase, (10) T303_07890 Galactokinase, (11) T303_09500 Fructokinase, (12) T303_06845 6-phosphofructokinase, (13) T303_02195 Phosphoglucose isomerase, (14) T303_05515 glucosamine-fructose-6-phosphate aminotransferase, (15) T303_07195 Phosphoglucosamine mutase, (16 & 17) T303_03955 N-acetylglucosamine-1-phosphate uridyltransferase (bifunctional), (18) T303_06336 UDP-galactopyranose mutase.

**Figure 6:**
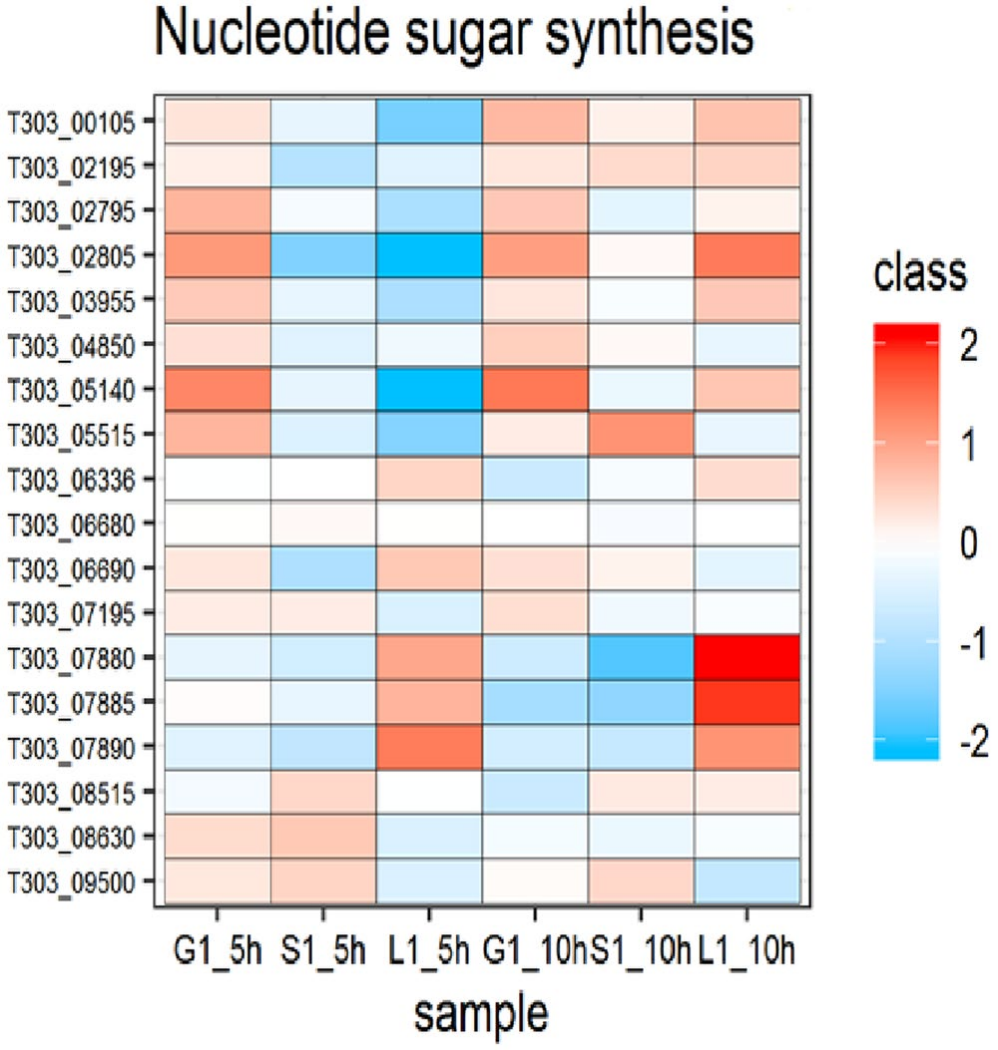
Changes in expression of proteins associated with nucleotide sugar synthesis. Heat map of DEPs involved in nucleotide sugar synthesis in the presence of glucose, sucrose and lactose at 5 h and 10 h. Red and blue box indicate up-regulated and down-regulated proteins, respectively.

**Figure 7:**
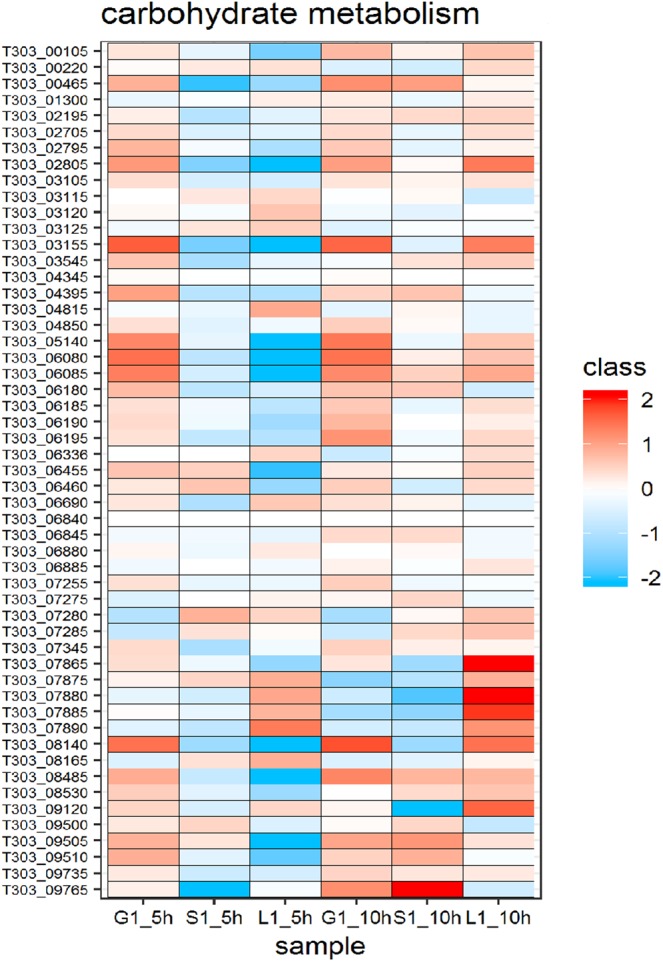
Changes in expression of proteins associated with carbohydrate metabolism. Heat map of DEPs involved in carbohydrate metabolism in the presence of glucose, sucrose and lactose at 5 h and 10 h. Red and blue box indicate up-regulated and down-regulated proteins, respectively.

Nucleotide sugars are important precursors in EPS biosynthesis. The pathway responsible for the formation of nucleotide sugars in *S. thermophilus* 1275 from various sugars is shown in Fig. 5. DEPs involved in sugar nucleotide formation in *S. thermophilus* 1275 in the presence of glucose, sucrose, lactose are shown in Figs. 6 and 7, Table 1 and Supplementary Table S1. In sucrose medium at 10 h (high EPS producing condition), UDP-galactose 4 epimerase (T303_06690) phosphoglucose isomerase (T303_02195), and glutamine-fructose-6-phosphate transaminase (T303_05515) were highly upregulated; β-galactosidase (T303_07865), galactose mutarotase (T303_07875) and galactose 1-phosphate uridyltransferase (T303_07885) were highly down-regulated. Other up-regulated proteins in M17-S at 10 h were glucokinase (T303_04850), UDP-glucose pyrophosphorylase (T303_00105), N-acetylglucosamine-1-phosphate uridyltransferase (T303_03955), and down-regulated proteins were 6-phosphofructokinase (T303_03155), phosphoglucosamine mutase (T303_07195) and UDP-galactopyranose mutase (T303_06336).

In M17-G at 10 h, two proteins in sugar nucleotide synthesis pathway galactose-1-phosphate uridyltransferase (T303_07885) and galactose mutarotase (T303_07875) were significantly downregulated. Other proteins involved in sugar nucleotide synthesis pathway showed non-significant changes. A non-significant downregulation was observed for β-galactosidase (T303_07865) and galactokinase (T303_07890), and a non-significant up-regulation was observed for UDP-glucose pyrophosphorylase (T303_00105), glucokinase (T303_04850), phosphoglucosamine mutase (T303_07195), phosphoglucomutase (T303_05140), phosphoglucose isomerase (T303_02195) and UDP-galactose 4 epimerase (T303_06690). Lactose media showed up-regulation of most of the proteins involved in nucleotide sugar formation. Proteins up-regulated in lactose media include 6-phosphofructokinase (T303_03155), galactose 1-phosphate uridyltransferase (T303_07885), UDP-glucose 4 epimerase (T303_07880), β-galactosidase (T303_07865), glutamine fructose 6 phosphate transaminase (T303_05515), N-acetylglucosamine-1-phosphate uridyltransferase (T303_03955), UDP-glucose pyrophosphorylase (T303_00105), phosphoglucomutase (T303_05140), phosphoglucosamine mutase (T303_07195), phosphoglucose isomerase (T303_02195). We observed down-regulation of proteins UDP-galactose 4 epimerase (T303_06690), galactokinase (T303_07890), glucokinase (T303_04850), UDP-galactopyranose mutate (T303_06336) and fructokinase (T303_09500) in M17-L at 10 h when compared to 5 h.

The proteins involved in other carbohydrate metabolism pathways were highly expressed other than EPS biosynthesis proteins at 10 h in the presence of selected sugars. A five-fold upregulation was observed in glyceraldehyde-3-phosphate dehydrogenase (T303_09765) in sucrose medium at 10 h. Other glycolytic proteins upregulated were phosphoglycerate kinase (T303_09735), lactose dehydrogenase (T303_04395), dihydrolipamide dehydrogenase (T303_06180) and fructose-1,6-bisphosphate (T303_00465). Formate acetyltransferase (T303_09120) involved in pyruvate metabolism was significantly downregulated. In lactose at 10 h, proteins involved in pentose phosphate pathway (phosphopentomutase T303_06455; ribose 5-phosphate isomerase, T303_06460; transketolase, T303_02705), glycolysis (6-phosphofructokinase, T303_03155; triosephosphate isomerase, T303_03545), pyruvate metabolism (phosphoenol pyruvate carboxylase, T303_04815; pyruvate dehydrogenase E2 component, T303_06185; pyruvate dehydrogenase E1 component, T303_06190; pyruvate dehydrogenase E2 component alpha subunit, T303_06195; phosphoacetyl transferase, T303_08140; formate acetyltransferase, T303_09120; lactoyl glutathionine lyase, T303_ 08485) and starch metabolism (α- amylase, T303_08530; glycogen phosphorylase, T303_06080; glucanotransferase, T303_06085) were upregulated. Downregulated proteins were those involved in fatty acid biosynthesis (acetyl-CoA carboxylase biotin carboxylase subunit, T303_03115; acetyl-CoA carboxylase subunit alpha, T303_03125), pyruvate metabolism (isopropylmalate synthase, T303_06880) and citrate cycle (isocitrate dehydrogenase, T303_07275). In glucose medium, there was no significant up/down regulation in other carbohydrate metabolism pathways at 10 h when compared to 5 h.

### EPS assembly

**Figure 8:**
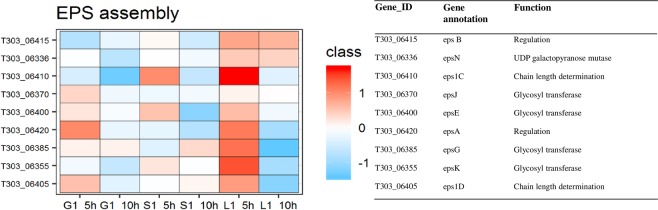
Changes in the expression of proteins associated with EPS assembly. (**a**) EPS gene cluster of *S. thermophilus* 1275. (**b**) Heat map of DEPs in EPS gene cluster in the presence of glucose, sucrose and lactose at 5 h and 10 h. Red and blue box indicate up-regulated and down-regulated proteins, respectively.

EPS gene cluster of *S. thermophilus* 1275 has 20 *eps* genes out of which 9 DEPs were observed in this study (Fig. 8, Table 1 and Supplementary Table S1). When DEPs at 10 h were compared with those at 5 h, *epsB* assigned for the regulation EPS biosynthesis was up-regulated in glucose medium, while *epsG*, *epsJ* assigned for glycosyl transferase and *eps1D* responsible for chain length determination was found to be up-regulated in sucrose medium. However, in lactose medium, six out of nine DEPs were found to be down-regulated. These include *epsA1C1DEGK* in which *epsA* involve in regulation, *eps1C1D*, which is responsible for chain length determination and *epsEGK* which is assigned for glycosyl transferase.

### EPS transport

The transport of EPS from cytoplasm to the external environment can occur through various pathways. In *S. thermophilus* 1275 Wzy/Wzx pathway was found to be responsible for the transport of EPS based on the expression of genes^13^. However, in the proteomic study flippase (*epsLM*) and polymerase proteins (*epsI*) involved in Wzy/Wzx pathway were not included in the DEPs (Fig. 8).

## Discussion

*S. thermophilus* 1275 was found to produce high amount of EPS in 1% sucrose supplemented medium when compared to lactose and glucose^13^. Hence, in this study, we investigated the proteome of high EPS producing dairy bacterium *S. thermophilus* 1275 and compared the proteomic level differences in this bacterium under the influence of three sugars, glucose, sucrose and lactose at two growth phases 5 h and 10 h. This is the first study providing insight into the proteomics of a high EPS producing lactic acid bacterium. The differential expression of 65 proteins suggested a significant change in the global response in *S. thermophilus* 1275 in sucrose media (Fig. 1). Among these many of the DEPs contributed to the steps involved in amino sugar and nucleotide sugar synthesis which are precursors for EPS biosynthesis (Supplementary Table S1). Lee *et al*.^17^ has performed genomic and proteomic analysis in *Sphingobium chungbukense* DJ77 for finding evidences of EPS biosynthesis.

Initial step in EPS biosynthesis is the transport of sugars from the external environment into the cytoplasm^16^. The general transport systems found in *S. thermophilus* 1275 are group translocation systems, primary transport systems and secondary transport systems^18^. Group translocation systems are dependent on phosphoenolpyruvate (sugar) phosphotransferase (PEP-PTS). Sugars are phosphorylated to sugar-6-phosphates during the transport through PEP-PTS. The proteins involved in PEP-PTS transport was found to be up-regulated in *S. thermophilus* 1275 (Fig. 4). Glucose transport was performed by PTS mannose transporters and sucrose transport was performed PTS sucrose transporters as indicated by the up-regulation of proteins responsible for PTS mannose transporter IIAB and IID as well as PTS sucrose transporter subunit IIABC, respectively (Fig. 4, Table 1 and Supplementary Table S1). Multi-functional PTS mannose system was found to transport glucose in many streptococci, *E. coli* and *Lactococcus lactis*^19–23^. Lactose transport is through lactose permease (*lacS*) as reported in our previous studies^13,24^. More than a two-fold increase in protein expression was observed at 10 h in lactose medium. We observed upregulation of PTS sucrose and PTS mannose subunits in M17-L medium where lactose was the only sugar source. This is probably due to the effort put forward by the starved cell while searching for alternative energy source^25^ as lactose depleted in the medium after 6 h (Supplementary Fig. S1). Poolman *et al*.^26^ also reported the involvement of sucrose PTS system in lactose transport at stationary phase with the help of HPr. The results obtained for the sugar transport system of *S. thermophilus* 1275 in the presence of glucose, sucrose and lactose were consistent with our mRNA level expression studies^13^.

EPS biosynthesis occurs after the formation of amino sugar and nucleotide sugar precursors^27^. Therefore, we closely analysed the nucleotide sugar formation pathway and proteins involved in the formation of nucleotide sugars in *S. thermophilus* 1275 under the influence of glucose, sucrose and lactose (Figs. 5–8) as this can be utilised to engineer strains that has an increased flux towards EPS biosynthesis^28^. Phosphorylation of the sugars transported into the cytoplasm determines its fate. Nucleotide sugars that lead to EPS biosynthesis are formed from sugar-1-phosphates, while sugar-6-phosphates enters glycolytic pathway^18^. In *S. thermophilus* 1275, glucose and sucrose are transported into the cell using PEP-PTS transporters that lead to the formation of sugar-6-phosphates. One of the key enzymes that determine the fate of sugar-6P either to enter glycolysis or EPS biosynthesis is phosphoglucomutase^29^. In the presence of all the three selected sugars, phosphoglucomutase (T303_05140) was upregulated at tenth hour when compared to fifth hour. Around three-fold upregulation was observed in lactose medium (Supplementary Table 1). However, due to the lack of lactose in the medium after 6 h (Fig. S1), EPS production was very less in this medium when compared to other sugars. However, in sucrose medium and glucose medium, the upregulation of this protein was insignificant at 10 h. This might be due the difference in the rate of metabolism and expression of genes in the presence of different sugars.

In sucrose medium at 10 h, UDP - glucose pyrophosphorylase (T303_00105) that leads to the formation UDP-glucose and UDP-galactose-4-epimerase (T303_06690) that leads to the formation of UDP galactose/glucose were found to be significantly upregulated. Phosphoglucose isomerase (T303_02195) that interchangeably convert fructose-6P to glucose-6P, glucosamine-fructose-6-phosphate aminotransferase (T303_05515), the first enzyme that leads to the production of UDP-GlcNAc were also significantly upregulated at tenth hour in sucrose medium. UDP - glucose pyrophosphorylase and phosphoglucose isomerase upregulation were also observed at mRNA level^13^.However, the expression of UDP-galactopyranose mutase gene (T303_06336) which was highly expressed in mRNA level were not observed in protein level. In M17-G at 10 h, glucokinase (T303_04850 - converts glucose to glucose-6P), phosphoglucomutase - T303_05140 (converts glucose-6P to glucose-1P) and UDP - glucose pyrophosphorylase - T303_00105 (converts glucose-1P to UDP-glucose) were upregulated which leads to nucleotide sugar production and finally EPS assembly (Fig. 7). In addition to this, glycolysis was occurring in parallel for the energy production and survival of the bacterium. Reports show that enzymes related to sugar nucleotide synthesis especially UDP-glucose pyrophosphorylase, UDP-galactose-4-epimerase and phosphoglucomutase are linked to increased EPS synthesis^30–32^.

Lactose is transported into the cell without phosphorylation due to the involvement of lactose permease which functions as symport. Then, β-galactosidase (T303_07865) cleaves lactose into glucose and galactose. The enzyme galactose 1-phosphate uridyltransferase (T303_ 07885) which converts galactose-1P to glucose-1P was upregulated in lactose medium at 10 h. The enzymes responsible for the formation of UDP-glucose and UDP-galactose were also found to be upregulated in lactose medium. In both sucrose and lactose medium, enzymes that lead to the formation of the nucleotide sugar UDP-GlcNAc were active at 10 h. From the previous transcriptomics study^13^ and current proteomic study, it is evident that the major nucleotide sugars involved in EPS biosynthesis in *S. thermophilus* 1275 were UDP-glucose and UDP-galactose. However, in a previous study mannose was also identified as a monomer in the EPS produced by *S. thermophilus* 1275^33^. This can be due to the mannose derived from the medium used for the study^34,35^.

Another key feature under consideration while dealing with EPS biosynthesis is the *eps* gene cluster. Generally, *eps* gene clusters in LAB genomes are highly distinct and the nucleotide sequences are very diverse. From the 51 *S. thermophilus* strains known, around 21 diverse *eps* gene clusters have been identified^18^. In this high EPS producing *S. thermophilus* 1275 we identified a unique *eps* gene cluster with two sets of genes *epsC* and *epsD* that codes for chain length determination^10^. The expression of proteins involved in *eps* gene cluster was found to be distinct in each sugar under each growth phase (Fig. 8). Out of 20 genes in the *eps* gene cluster of *S. thermophilus* 1275, we observed the expression of 9 proteins in this study. However, in mRNA level all the 20 genes in *eps* gene cluster was expressed. Haider and Pal^36^ reported that this low protein expression level when compared to mRNA transcripts can be due to the diversity in post transcription machinery and dissimilar half-lives.

Among the 9 genes expressed in the *eps* gene cluster of *S. thermophilus* 1275, regulatory gene *epsB* was upregulated at 10 h in glucose medium. All the 9 DEPs were downregulated in lactose medium indicating the decreased level of EPS production at 10 h. However, glycosyl transferase (*epsG*) and chain length determining protein (*eps1D*) were found to be active in sucrose medium at 10 h, the high EPS producing condition, when compared to sucrose medium at 5 h. In *Lactobacillus fermentum* TDS030603, elevated expression of *epsB*, *epsE* and *epsG* was observed at high EPS producing condition in a chemically defined medium^37^. Similarly, overexpression of *epsD*, priming glucosyltransferase, in *L. lactis* NIZO B40 resulted in increased EPS production^38^. High EPS production was observed in *L. lactis* during the overexpression of complete *eps* gene cluster^39^. Hence, to increase EPS production overexpression of *eps* gene cluster can be a useful approach^40^.

In conclusion, we compared the expression of proteins in high EPS producing *S. thermophilus* 1275 in the presence of three sugars, glucose, sucrose and lactose at log phase (5 h) and stationary phase (10 h) using iTRAQ labelling and LC/MS/MS, with focus on proteins involved in EPS biosynthesis and transport. It was identified that PEP-PTS transport system was involved in the transport of sucrose and glucose into the cytoplasm of *S. thermophilus* 1275 while *lacS* was responsible for lactose intake. UDP-glucose and UDP-galactose were the major sugar nucleotide precursors formed in the presence of each sugar. Glycosyl transferase and chain length determining proteins were found to be overexpressed in the presence of sucrose, that led to high EPS production. Moreover, an over expression of proteins linked with arginine metabolism and alanine/aspartate and glutamate metabolism was also observed at elevated EPS production. Thus, this work provides an insight into the major proteins and related pathways involved in high EPS producing conditions in *S. thermophilus* 1275.

## Materials and Methods

### Bacterial strain and fermentation conditions

*S. thermophilus* ASCC 1275 procured from Dairy Innovation Australian Limited was stored in M17 broth (BD Company, Franklin Lakes, NJ, USA) containing 20% (v/v) glycerol at −80 °C. The bacterium was activated using 1% inoculum once in M17 broth at 37 °C for 18 h before use. Based on the KEGG pathway analysis of *S. thermophilus* 1275 and preliminary screening, three sugars, glucose (G), lactose (L) and sucrose (S) were selected for the study. After the initial activation, 1% inoculum was again activated in M17 broth containing 1% of the selected sugars as the sole carbon source, i.e. M17-glucose (M17-G), M17-lactose (M17-L) and M17-sucrose (M17-S). Later, the bacterium was transferred into a stirred reactor (GLS 80^®^ - Duran group, Mainz, Germany) with 1 L M17 fermentation broth containing the sugar (at 1% concentration) in which it was activated previously. Fermentation was performed at 37 °C for 24 h and 10 mL samples were collected at 5 h (log phase) and 10 h (stationary phase) for iTRAQ proteomic analysis. Bacterial cells were collected by centrifugation for 15 min at 10,000 × g and stored at −80 °C. For each sugar, the fermentation experiment was performed in triplicates.

### Estimation of growth, pH, residual sugar and lactic acid production

Growth curve, pH, residual sugar and lactic acid production of *S. thermophilus* 1275 were analysed under the influence of glucose, lactose and sucrose. Samples (3 mL) were collected over a period of 0 h to 24 h at every 6 h. SmartSpec™ Plus Spectrophotometer (Bio-Rad Laboratories, Hercules, CA, USA) was used to analyse the growth curve and Orion Model 250 A portable pH meter (Thermo Fischer, Waltham, MA, USA) was used for pH estimation. Residual sugar analysis was performed using high performance liquid chromatography (HPLC). For this, 1 mL sample was centrifuged for 10 min at 10,000 × g and the supernatant collected was diluted 10 times using sulphuric acid (5 mM). Simultaneous detection of residual sugar and lactic acid production were performed using LC- 2010A (Schimadzu Corp., Kyoto, Kyoto Prefecture, Japan) coupled with refractive index detector and UV-Vis detector (220 nm), respectively, connected in series. An isocratic elution with sulphuric acid (5 mM) at 0.8 mL/min flow rate and 65 °C column temperature was performed using an anion exchange column HPX-87H (300 × 7.8 mm, 9 µm; Bio-Rad Laboratories, Hercules, CA, USA) to detect sugars and lactic acid.

### Protein extraction and precipitation

Extraction of protein from *S. thermophilus* 1275 cells was performed according to Wu *et al*.^14^. Bacterial cells stored at −80 °C were thawed on ice and resuspended in lysis buffer made of 50 mM Tris pH 8.4, 2 mM β- mercaptoethanol, 0.1% SDS (w/v), 150 mM NaCl and cOmplete^TM^ Mini Protease Inhibitor Cocktail (Roche, Basel, Switzerland) – one tablet yielded 1 mm EDTA in 10 mL. Total proteins were extracted from bacterial cells by sonication (Soniprep 150; Labtech, Heathfield, ES, UK) on ice for 45 cycles (10 sec ON, 10 sec OFF). Cell lysates were centrifuged at 4 °C for 15 min at 12000 × g and supernatant containing proteins were collected. Total protein concentration in each sample was estimated using Bradford assay with bovine serum albumin (BSA), dissolved in the above-mentioned lysis buffer, as the standard. From each sample stored at 4 °C, 100 µg of protein was precipitated using six volumes of acetone chilled at −20 °C. The tubes were incubated at −20 °C for 1 h and centrifuged at 6000 × g for 10 min. The pellets were collected and used for further analysis.

### iTRAQ-based peptide labelling and LC-MS/MS analysis

Acetone precipitated proteins were used for iTRAQ labelling as per the manufacturer’s instructions provided in the iTRAQ^®^ Reagents 8-plex kit (Applied Biosystems, Foster City, CA, USA). Briefly, protein reduction was performed by adding 20 µL dissolution buffer, 1 µL denaturant (2% SDS) and 2 µL reducing reagent to each tube containing 100 µg of protein. Samples were vortexed, span for 45 sec in a minicentrifuge (VWR International, Radnor, PA, USA) and incubated at 60 °C for 1 h. Then, cysteine blocking was performed by adding 1 µL of cysteine blocking reagent to each sample after spinning. Again, the tubes were vortexed, span and incubated at room temperature for 10 min. Cysteine blocked proteins were digested with 1 mg/mL trypsin (Sigma-Aldrich, St. Louis, MO, USA) with protein to trypsin ratio 50:1 at 37 °C for 16 h. Complete digestion of the proteins was verified using SDS-PAGE. Peptides obtained were purified using C18 Sep-Pak purification kit (Waters; Milford, MA, USA).

The purified peptides were used for iTRAQ labelling. Initially, 50 µL of isopropanol was added to each iTRAQ reagent vial at room temperature (25 °C). Samples were labelled with iTRAQ tags as follows: G (5 h)-113 tag; G (10 h): 114 tag; S (5 h): 115 tag; S (10 h): 116 tag; L (5 h): 117 tag; L (10 h): 118 tag. The purified peptides were used for iTRAQ labelling. Initially, 50 µL of isopropanol was added to each iTRAQ reagent vial at room temperature. Samples were labelled with iTRAQ tags as follows: G (5 h)-113 tag; G (10 h): 114 tag; S (5 h): 115 tag; S (10 h): 116 tag; L (5 h): 117 tag; L (10 h): 118 tag. After labelling the peptides with iTRAQ tags, they were incubated at room temperature for 2 h. The contents from each iTRAQ labelled tubes were combined and used for clean-up followed by fractionation using a high-resolution cation-exchange column.

The iTRAQ labelled peptides were dissolved in 5 ml solvent A (10 mM KH_2_PO_4_, 25% CAN; pH 3.0) and loaded onto polysulfoethyl A™ column (2.1 × 100 mm) with 5 µm particles (Poly LC, Columbia, MD, USA) after conditioning. The peptides were eluted at the flow rate of 0.2 ml/min with a gradient of solvent A for 0 min, solvent B (solvent A+ 350 mM KCl) for 0 min to 60 min, solvent B for 5 min, solvent A for 5 min, solvent A for 10 min. The samples were collected at every 1 min to monitor the absorbance at 220 nm with DAD detector. Eluted peptides were pooled into 16 fractions, vacuum dried and LC/MS/MS analysis was performed as previously described Bi *et al*.^41^ using Triple TOF 6600 system fitted with Nanospray III source (Sciex, Framingham, MA, USA).

### Proteomic data analysis and statistical testing

Data was analysed using the method described by Wu et al. (2019)^14^. Raw data files obtained from mass analyser were converted into MGF files using ProteinPilot 5.0.1.0. Proteins were identified using these MGF files by Mascot search engine against the protein translation database containing *S. thermophilus* 1275 sequences. Paragon algorithm was used to search mass spectrums against target-decoy database. Global and false discovery rate (FDR) analysis was performed using Proteomics System Performance Evaluation Software, an inbuilt feature in ProteinPilot. Proteins were filtered using a minimal unused ProtScore with at least two peptides having critical FDR 1% and confidence level above 95%. After filtering the proteomics data, the protein expression matrix was built by the relative expression level of each sample against the 113 channel. Then the expression for each protein was normalized and log 2 transformed by the mean value of all samples. The p-value and log2 fold change of differential expression genes were approached by the t-test between each sample group. DEPs were identified based on log FC = 0.58 and FC = 1.5 with p < 0.1 (two-tailed). The heatmap and volcano plot was generated by ggplot2 in R 3.4.4. The COG database (http://clovr.org/docs/clusters-of-orthologous-groups-cogs/) and KEGG database (https://www.genome.jp/kegg/) were used to classify the identified proteins.

### RNA Extraction and Real time-qPCR assay

*S. thermophilus* 1275 was cultured in M17 broth supplemented with glucose, sucrose and lactose as described above, and total RNA was extracted using Ambion RiboPure™ -Yeast kit as per manufacturer’s instructions. Contaminating DNA were removed using RNase-free DNase I. The purity and concentration of RNA was estimated using NanoDrop™ UV spectrophotometer (Thermo Scientific, DE, USA). The proteomic results were validated using StepOnePlus™ RT-qPCR system (Applied Biosystem, Foster City, CA, USA). Total eight genes were selected from all the conditions G10h Vs G5h, S10h Vs S5h and L10h Vs L5h (Supplementary Table S7). cDNA was synthesized from the RNA using High-Capacity RNA-to-cDNA™ Kit (Applied Biosystems). qPCR was performed using the gene-specific primers (Supplementary Table S7), cDNA from different conditions and SYBR Green Master Mix. The reactions were incubated at 95 °C for 5 min; 40 cycles at 95 °C for 10 s; 55 °C for 30 s and 72 °C for 20 s. The reference gene used was *tuf* gene. Comparative critical threshold method (2^− ΔΔCt^) was used to calculate the relative expression of each target gene^42^.

## Supplementary information

Supplementary figure S1.

Table S1.

Table S2.

Table S3.

Table S4.

Table S5.

Table S6.

Table S7.

## Data Availability

The authors confirm that the data supporting the findings of this study are available within the article [and/or] its supplementary materials.
