# Supplementary material for: Proteomic analysis reveals potential factors associated with enhanced EPS production in Streptococcus thermophilus ASCC 1275
Source: Sci Rep. 2020 Jan 21;10:807. doi: 10.1038/s41598-020-57665-9 (PMC6972726; doi:10.1038/s41598-020-57665-9)
Supplement: Supplementary file 1 — Supplementary figure S1. [file 41598_2020_57665_MOESM1_ESM.docx]

**Supplementary Information**

**Proteomic analysis reveals potential factors associated with enhanced EPS production in *Streptococcus thermophilus* ASCC 1275**

Aparna Padmanabhan, Yin Tong, Qinglong Wu, Clive Lo, Nagendra P. Shah

**Supplementary figure S1.** EPS production, sugar utilization and lactic acid production of *S. thermophilus* 1275 in M17 media supplemented with 1% glucose, 1% sucrose and 1% lactose supplemented at different time points^13^. a) Amount of EPS produced by *S. thermophilus* 1275 b) Glucose utilization and lactic acid production c) Sucrose utilization and lactic acid production d) lactose utilization and lactic acid production.
